# Cathepsin L3 From *Fasciola hepatica* Induces NLRP3 Inflammasome Alternative Activation in Murine Dendritic Cells

**DOI:** 10.3389/fimmu.2019.00552

**Published:** 2019-03-22

**Authors:** Daiana Pamela Celias, Ileana Corvo, Leonardo Silvane, José Francisco Tort, Laura Silvina Chiapello, Manuel Fresno, Alicia Arranz, Claudia Cristina Motrán, Laura Cervi

**Affiliations:** ^1^Departamento de Bioquímica Clínica, Facultad de Ciencias Químicas, Universidad Nacional de Córdoba, Córdoba, Argentina; ^2^Centro de Investigaciones en Bioquímica Clínica e Inmunología (CIBICI), CONICET, Córdoba, Argentina; ^3^Laboratorio de Investigación y Desarrollo de Moléculas Bioactivas, CENUR Litoral Norte – Sede Paysandú, Universidad de la República, Paysandú, Uruguay; ^4^Departmento de Genética, Facultad de Medicina, Universidad de la República, Montevideo, Uruguay; ^5^Centro de Biología Molecular Severo Ochoa (CSIC), Madrid, Spain

**Keywords:** cathepsin L3, *Fasciola hepatica*, dendritic cells, NLRP3 inflammasome, IL-1β

## Abstract

The production of IL-1-family cytokines such as IL-1β and IL-18 is finely regulated by inflammasome activation after the recognition of pathogens associated molecular pattern (PAMPs) and danger associated molecular patterns (DAMPs). However, little is known about the helminth-derived molecules capable of activating the inflammasome. In the case of the helminth trematode *Fasciola hepatica*, the secretion of different cathepsin L cysteine peptidases (FhCL) is crucial for the parasite survival. Among these enzymes, cathepsin L3 (FhCL3) is expressed mainly in the juvenile or invasive stage. The ability of FhCL3 to digest collagen has demonstrated to be critical for intestinal tissue invasion during juvenile larvae migration. However, there is no information about the interaction of FhCL3 with the immune system. It has been shown here that FhCL3 induces a non-canonical inflammasome activation in dendritic cells (DCs), leading to IL-1β and IL-18 production without a previous microbial priming. Interestingly, this activation was depending on the cysteine protease activity of FhCL3 and the NLRP3 receptor, but independent of caspase activation. We also show that FhCL3 is internalized by DCs, promoting pro-IL-1β cleavage to its mature and biologically active form IL-1β, which is released to the extracellular environment. The FhCL3-induced NLRP3 inflammasome activation conditions DCs to promote a singular adaptive immune response, characterized by increased production of IFN-γ and IL-13. These data reveal an unexpected ability of FhCL3, a helminth-derived molecule, to activate the NLRP3 inflammasome, which is independent of the classical mechanism involving caspase activation.

## Introduction

Fasciolosis, caused by the helminth parasite *F. hepatica* is a chronic disease that affects the liver of cattle all over the world. It is estimated that this disease causes huge annual economic losses in livestock, due to a reduction in the production of milk, wool and meat in cows and sheep (1, 2). In addition, the World Health Organization (WHO) has reported that ~2.4 million people are infected by this parasite worldwide; and fasciolosis has recently been declared as an emerging disease in humans with an increased number of cases in some regions of the planet (3, 4). Mammals (human and cattle) are infected by the ingestion of the metacercariae, a parasitic form encystic in aquatic plants. At the early stage after infection, the parasites excyst in the small intestine. Then, juvenile flukes called newly excysted juveniles penetrate through the host intestine wall and migrate across the peritoneal cavity, until they reach the liver, which is the target organ of the infection (5).

*F. hepatica* has developed strategies for the evasion of the host immune response. It has been determined that different antigenic preparations of this parasite such as total extract (TE), *F. hepatica* tegumental antigen (FhTeg) and excretory-secretory products (ESP) decrease the activation state of DCs, induced by LPS (6–8). It has been established that the *F. hepatica* proteins cathepsin L1 (FhCL1), gluthation transferase (FhGST), and Kunitz type molecule (FhKTM) have a modulating effect on DCs, which leads to the suppression of the adaptive immune responses Th1 and/or Th17 (9, 10). In line with this, in our laboratory, it has been demonstrated a correlation between PDL-2 expression in macrophages and the promotion of Th2 response, which in turn inhibits Th1 profile during infection with *F. hepatica* (11). These results suggest that there would be molecules within the products derived from the parasite, that promote inflammatory type responses. According to this hypothesis, data from other authors have demonstrated that a mucin-like peptide derived from *F. hepatica* has immunostimulatory properties, increasing the ability of DCs to promote IFN-γ responses in allogeneic splenocytes (12).

Despite the existence of these parasite molecules that promotes inflammatory responses, the parasite infection mainly induces Th2 and T reg cells, suppressing a Th1 type response. However, an exacerbated Th2 response could also be deleterious for the parasite.

In a recent study, the chronicity of helminth infection has been related to the ability of the intestinal parasite *Heligmosomoides polygyrusbakeri (Hp)* to induce IL-1β secretion, which in turn inhibits IL-33 and IL-25, resulting in the control of an excessive Th2 response (13).

It is well-known that two signals are required for inflammasome-dependent IL-1β cytokine production. The first signal involves cellular activation by microbial stimuli or endogenous danger signals which activate pattern recognition receptors such as the Toll-like receptor (TLR), leading to the activation of the transcription factor NF-κB and consequently the synthesis of pro-IL1β and components of the inflammasome complex. The second signal is the activation of the inflammasome multiprotein complex by different stimuli such as ATP, silica, amyloid-β peptide, among others, that leads to the autocatalysis of pro-caspase-1, which cleaves the pro-IL-1β into bioactive IL-1β form (14).

Among the inflammasomes, the pyrin domain containing nod-like receptor 3 (NLRP3) inflammasome, is the most studied because it plays a major role in the immune response against different pathogens such as viruses (15–17), bacteria (18), protozoan parasites (19), and fungal pathogens (20, 21). However, the role of inflammasome after helminth product stimulation or helminth infection, is less clear so far. In relation to this, there is a report showing that soluble eggs antigen (SEA) from *Schistosoma mansoni* induces the inflammasome activation, increasing IL-1β secretion, after TLR priming on dendritic cells (DCs) (22). In contrast, a secreted peptide from *F. hepatica*, the helminth defense molecule 1 (FhHDM-1) prevents NLRP3 activation and IL-1β production (23).

As regards the molecules secreted by *F. hepatica* during its migration, cathepsin L family, represents 80% of the peptidases with cysteine protease activity. Cathepsins are critical for the parasite survival, since they are able to digest the protein contents of the blood, including haemoglobin, albumin, and immunoglobulin. FhCL1 and FhCL2 are predominant in mature stages, while FhCL3 is mainly expressed in the juvenile form or invasive stage (24, 25). Although both, FhCL2 and FhCL3 have shown collagenolytic activity, FhCL3 has demonstrated greater ability to digest type I and II collagens compared to FhCL2. It has been reported that FhCL1 induces some pro-inflammatory mediator secretion such as IL-6, IL-12p40 (26) and macrophage inflammatory protein 2 (MIP-2) with enhanced CD40 expression in DCs (9). In addition, these immunomodulatory functions were shown to be dependent on TLR4 and on its cystein protease activity (9). Although FhCL3 is an important part of the proteolytic machinery of *F. hepatica*, its interaction with the immune system is unknown so far.

In the present study, we investigate the capacity of an active recombinant FhCL3 to modulate murine DC functions. We show an unexpected ability of FhCL3 to induce IL-1β and IL-18 secretion by DCs, which was dependent on its enzymatic activity and NLRP3 activation. Surprisingly, the lack of priming as well as caspase1/11 requirement for IL-1β production by FhCL3-treated DCs, indicate that alternative inflammasome activation mechanisms are carried out by this parasite molecule. Here we demonstrate that FhCL3 is internalized and targeted to the cytosol, where the pro-IL-1β cytokine is cleaved to its mature and biologically active form. Besides, FhCL3-dependent NLRP3 inflammasome alternative activation was associated with reactive oxygen species (ROS) production, lysosomal acidification and potassium channel efflux. Finally, we show that the interaction of DCs with FhCL3 has consequences on adaptive immune response, since it confers a unique expression pattern of cytokines IFN-γ and IL-13 that could be protective in this parasitosis or in other helminth infections.

## Materials and Methods

### Mice

Six- to eight-week-old inbred female C57BL/6 mice indicated as wild type (WT) along the manuscript, were obtained from the Faculty of Veterinary Sciences, National University of Litoral (UNL, Argentina). CASP1/11 KO (B6N.129S2-Casp1^tm1Flv^/J) and NLRP3 KO (B6.129S6-Nlrp3/^Jtm1Bhk^) mice, on a C57BL/6 background, were purchased from Jackson Laboratory (Bar Harbor, ME, USA). The BALB/c mice used were obtained from the Comisión Nacional de Energía Atómica (Buenos Aires, Argentina).

All animal experiments were approved by and conducted in accordance with guidelines of the committee for Animal Care and Use of the Faculty of Chemical Sciences, National University of Córdoba (Approval Number HCD 1637) in strict accordance with the recommendation of the Guide to the Care and Use of Experimental Animals published by the Canadian Council on Animal Care (OLAW Assurance number A5802-01).

### Production of FhCL3 and rvFhCL3

Active FhCL3 and recombinant variant rvFhCL3 were produced in the yeast *Hansenula polymorpha* as previously described (25, 26). rvFhCL3 has a Trp67Leu substitution which drastically reduces enzyme activity toward P_2_-Pro and P_3_-Gly substrates and renders the enzyme unable to digest type I collagen (27). Recombinant pro-peptidases were secreted to the culture media, and recovered by 20–30 fold concentration of culture supernatants by ultrafiltration with a 10 kDa cut-off membrane. The pro-enzymes were auto-catalytically activated to the mature form by incubation for 2 h at 37°C in 0.1 M sodium citrate buffer (pH 5.0) with 2 mM DTT and 2.5 mM EDTA, dialyzed against PBS pH 7.3 and stored at −20°C. The protein concentration was assessed by the bicinchoninic acid (BCA) method (27). Endotoxins were removed by using polymyxin B columns (Thermo Fisher Scientific, Waltham, MA, USA), according to the manufacturer's instructions. The presence of endotoxins was assessed before and after removing endotoxins using the Chromogenic Limulus Amebocyte Lysate QCL-1000 Assay (Lonza, Walkersville, MD, USA) following the manufacturer's instructions. Both proteins (active FhCL3 and rvFhCL3), after polymyxn B columns, were revealed to have endotoxin levels similar to those of background and complete RPMI 1,640 medium (supplemented with 5% heat-inactivated fetal bovine serum, 40 μg/ml gentamicin, 2 mM L-glutamine, and 50 μM 2-mercaptoethanol), so they were taken to be endotoxin free (data not shown).

### FhCL3 Enzymatic Activity Characterization

The FhCL3 used in this study is a functionally active recombinant protein (Length: 226 aa, Mass (kDa): 26) expressed in yeast and isolated as previously described (25, 28). FhCL3 exhibited an enzyme activity detected by a fluorescence assay using the synthetic fluorogenic peptide Tos-GPR-AMC. When this peptide is hydrolyzed it releases the fluorescent group 7-amino-4-methyl coumarin (AMC). The measurement of fluorescence (RFU) was performed at 60-s intervals for 20 min (Supplementary Figure 1A). Furthermore, the analysis of recombinant FhCL3 by The MALDI-TOF mass spectrum, revealed two peaks: one, the most abundant of 29 kDa and another of 38.6 kDa corresponding to the expected MW values for the mature enzyme and pro-enzyme, respectively (Supplementary Figure 1B).

### DCs Generation and Stimulation

DCs were generated as previously described, with slight modifications (6). Mice were sacrificed by cervical dislocation, and bone marrow was collected from femurs and tibiae of mice, and the cells were cultured with 7.5% of supernatant from GM-CSF, which produced J558 cells (20 ng/ml final concentration in the plate) in 8 days. After this time period, the expression of CD11c and MHCII were quantified by flow cytometry using FITC- or PE-conjugated Ab purchased from BD Biosciences, San Jose, CA, USA. Harvested cells comprised between 75 and 90% DCs (MHCII+, CD11c+). DCs viability was evaluated by 3-(4,5-dimethylthiazol-2-yl)-2,5-diphenyltetrazolium bromide (MTT) assay (Sigma-Aldrich St. Louis, MO, USA) after all treatments it was about 90%. Besides, Lactate dehydrogenase (LDH) release was measured by a colorimetric assay kit (Wiener Lab) according to the manufacturer's protocol and FhCL3 treatment did not induce cytotoxicity in DCs.

### Stimuli and Inhibitors Treatments

At day 8 of differentiation, DCs were treated with FhCL3 (10 μg/ml), rvFhCL3 (10 μg/ml), LPS from *E. coli* serotype 055:B5 (100 ng/ml; Sigma-Aldrich,St. Louis, MO, USA) for 18 h and/or ATP (5 mM; Sigma-Aldrich St. Louis, MO, USA) the last 30 min of culture. In some cases, cells were pretreated with different inhibitors: a pan caspase inhibitor, carbobenzoxy-valyl-alanyl-aspartyl-[O-methyl]- fluoromethylketone (Z-VAD-FMK, 20 μM, during 1 h), a NADPH oxidase inhibitor, diphenyleneiodonium chloride (DPI, 20 μM, for 3 h), KCl (50 mM, for 90 min), chloroquine (5 mM for 1 h), all reagents were purchased in Sigma-Aldrich St. Louis, MO, USA. After pretreatment, the DCs were washed. Then, the cells and supernatants were collected for performing different techniques. The number of cells used for the different techniques was: 4 × 10^5^ cells/well for ELISA and Flow Cytometry, and 1 × 10^6^ cells/well for qPCR, Western blot and Confocal Microscopy.

### Flow Cytometry

After the FhCL3 and LPS treatments as described above, the expression of surface molecules on DCs was quantified by flow cytometry using anti-CD11c, anti-MHCI, anti-MHCII, anti-CD40, anti-CD80, and anti-CD86, all purchased from (BD Bioscience, San Jose, CA, USA). For the exclusion of dead cells, we used the Zombie Aqua fixable viability kit (BioLegend, San Diego, CA, USA). Samples were collected using the FACSCanto II and FACS LSR Fortessa (BD Biosciences, San Jose, CA, USA) and data were analyzed using the Flow Jo Software.

### Cytokine Measurement

IL-6, IL-12p70, TNF, IL-10, IL-4, and IFN-γ were purchased from BD Pharmingen, San Jose, CA, USA; IL-1β, IL-18, and IL-13 from Thermo Fisher Scientific, Waltham, MA, USA and IFN-β from BioLegend, San Diego, CA, USA. All cytokines were measured in culture supernatants by capture ELISA following the manufacturer's guidelines.

### Western Blot

Conditioned DCs as described above were treated with RIPA buffer, analyzed by 10% SDS-PAGE and transferred to a nitrocellulose membrane. After blocking with 5% milk, the membranes were incubated overnight at 4°C with primary rabbit and mouse monoclonal antibodies: anti-IκB-α Antibody (Cell Signaling Technology, Danvers, MA, USA), anti NF-kB p65 Polyclonal Antibody (ThermoFisher Scientific Waltham, MA, USA), and Anti-β-actin (Abcam, Cambridge, UK). β-actin was used as loading control. The sheets were revealed by incubation with corresponding IRD Fluor 800-labeled IgG or IRD Fluor 680-labeled IgG secondary antibodies (LI-COR Inc., Lincoln, NE, USA) for 1 h at room temperature. After washing, the membranes were scanned with the Odyssey CLx Infrared Imaging System (LI-COR, Lincoln, NE, USA) at a wavelength of 700–800 nm. Densitometric analysis was performed using FIJI/ImageJ software.

### Confocal Microscopy

FhCL3 were labeled with an Alexa Fluor 488 labeling protein kit (Molecular Probes- Thermo Fisher Scientific, Waltham, MA, USA) (29). DCs were incubated with Alexa Fluor 488-labeled-FhCL3 for 2, 4, or 18 h in RPMI complete medium. Then, the cells were fixed by incubation with 4% paraformaldehyde and subsequently permeabilized with 0.1% Triton-X/PBS. After this, cells were incubated for 1 h at room temperature with primary antibody specific for early endosome antigen, EEA-1 (Abcam, Cambridge, UK) or LAMP-1/CD107a (R&D Systems, MN, USA) diluted in 1% FBS/PBS. Following three washes with 1X PBS, the cells were incubated with secondary antibody, Alexa Fluor 647-conjugated Goat anti-Rabbit IgG (Thermo Fisher Scientific, Waltham, MA, USA) or Northern Lights 557–conjugated Goat anti-Rat IgG (R&D Systems, MN, USA) and then stained with Hoechst (1 μg/ml in PBS) to detect cell nuclei. Cells were examined either confocal microscope (Olympus FV1200), and images were analyzed using FIJI/ImageJ software.

### Quantitative Real-Time-PCR (q-PCR)

RNA was extracted from DCs by the Trizol reagent (Invitrogen, ThermoFisher Scientific, Waltham, MA, USA) and reverse-transcribed into cDNA by using Revert Aid First Strand cDNA Synthesis (Fermentas- ThermoFisher Scientific, Waltham, MA, USA). Transcripts were quantified by real-time quantitative PCR on an StepOnePlus™ Real-Time System using SYBR Green (ThermoFisher Scientific Waltham, MA, USA) with the following primers (all primers listed in the 5′ to 3′ orientation): *Il-1b* GCAACTGTTCCTGAACTCAACT (forward) and ATCTTTTGGGGTCCGTCAACT (reverse), *Actb* CGCCACCAGTTCGCCATGGA (forward) and TACAGCCCGGGGAGCATCGT (reverse). Relative expression was calculated using Delta-*Cq* method (RQ = E^−(Δ*Cq*)^), and normalized to the level of unstimulated DCs. The cycling conditions included a hot start at 95°C for 10 min, followed by 40 cycles at 95°C for 15 s and 60°C for 1 min.

### Direct Effects of FhCL3 on Recombinant Pro-IL-1β

Murine IL-1β standard (Thermo Fisher, Scientific Waltham, MA, USA) at a concentration of 500 pg/ml and murine pro-IL-1β standard (Thermo Fisher, Scientific Waltham, MA, USA) at a concentration of 1,000 pg/ml were mixed separately with FhCL3 (1 μg/ml) or rvFhCL3 (1 μg/ml) in a reaction buffer containing 0.1 M sodium phosphate buffer, pH 6.0, 1 mM DTT and 1 mM EDTA. The samples were kept in the incubator for 2 h, at 37°C. Then, the determination of mature IL-1β was performed by capture ELISA and Western blot. For ELISA, an IL-1β Mouse Uncoated ELISA Kit (Thermo Fisher, Scientific Waltham, MA, USA) was used. For western blot anti-IL-1β antibody (Cell Signaling Technology, Danvers, MA, USA) and a mouse IL-1 beta /IL-1F2 Antibody (R&D Systems, MN, USA) were used as primary antibodies, to detect IL-1β and pro-IL-1β, respectively. IRD Fluor 800-labeled IgG (LI-COR Inc., Lincoln, NE, USA) was as and secondary antibody.

### ROS Production

To evaluate the intracellular ROS, cytoplasmic ROS (cROS) and mitochondrial (mROS), DCs from each experimental condition were collected and washed with PBS 2% FBS. First, cells were stained with APC labeled anti-CD11c (BioLegend) and with PerCP labeled anti-MHCII (BD Pharmigen, San Diego, CA) antibodies for 20 min at 4°C. After that, DCs were incubated with 5 μM of a MitoSOX TM Red probe (Thermo Fisher, Scientific Waltham, MA, USA) for mROS or 20 μM H2DCFDA probe (Sigma Aldrich, St. Louis, MO, USA) for cROS detection for 20 min at 37°C. Finally, these cells were collected using the FACSCanto II (BD San José, CA, USA), and data were analyzed using the FlowJo Software.

### Allogeneic Mixed Lymphocyte Reaction

DCs from C57BL/6 mice (2 × 10^4^ cells/well) unstimulated or treated with FhCL3 (10 μg/ml) were cultured for 18 h. Collected cells were cultured with splenocytes from BALB/c mice (2 × 10^5^ cells/well) 0(10). After 5 days of culture, cells were collected, stimulated with PMA/Ionomycin for 4 h and analyzed by flow cytometry. While, the supernatants were analyzed for IFN-γ, IL-4, and IL-13 production by ELISA.

### *In vivo* DCs Treatment

DCs from C57BL/6 mice were treated for 18 h as described above, washed twice with PBS, and injected intraperitoneally in C57BL/6 mice (1 × 10^6^ cells/mice). After 7 days, inguinal lymph nodes (iLN) were recovery. Suspensions of lymphocytes were adjusted to 5 × 10^5^ cells/mL and cultured in 96-well plates in medium alone or in the presence of 10 μg/mL of FhCL3 for 48 h. Then, the cells were stimulated with PMA/Ionomycin for 4 h and analyzed by flow cytometry. For IFN-γ, IL-4, and IL-13 detection by ELISA, supernatans were collected after 48 h of FhCL3 re-stimulation.

### Statistical Analysis

The measurement of cytokines in the DCs supernatants was performed for three wells per culture, with 3 to 5 cultures being included in each group and the data expressed as means ± standard deviation. In some experiments data were analyzed by one or two-way ANOVA followed by Tukey or Bonferroni's post-test for comparing more than two groups. GraphPad Prism versión 6.01 was used for statistical comparisons. *P*-values of ^*^ < 0.05, ^**^ < 0.01 or ^***^ < 0.001 were considered to be statistically significant, depending on the experiment.

## Results

### FhCL3 Induces IL-1β and IL-18 Production Depending on its Cysteine Protease Activity

Despite the evidences showing that different molecules from *F. hepatica*, such as cysteine protease cathepsin L1 (FhCL1), glutathione transferase (FhGST), and Kunitz type molecule (KTM) are able to regulate DCs activation (9, 10), there is no information about the capacity of FhCL3, which is expressed in the invasive stage of this parasite, to modulate DCs functions.

**Figure 1:**
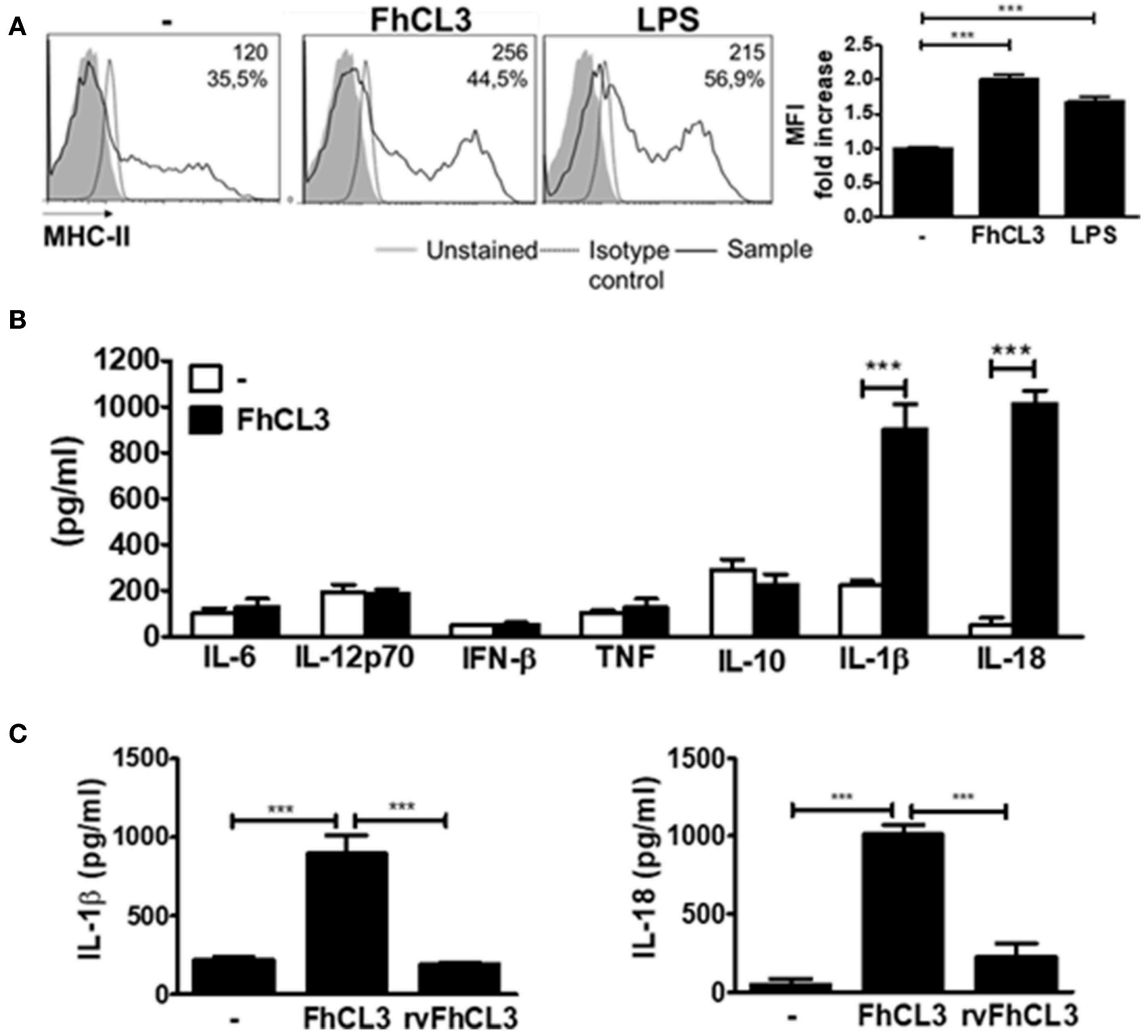
Active FhCL3 induces IL-1β and IL-18 production in DCs. **(A)** DCs from C57BL/6 mice were incubated with medium, FhCL3 (10 μg/ml) or LPS (100 ng/ml) for 18 h. Representative histograms show the cell surface expression of MHCII. Unstained cells (gray histograms), isotype controls (dotted lines), and FhCL3 or LPS-stimulated cells (black lines). Values represent the mean fluorescent intensity (MFI) and the percentage of positive cells. Bar graphs depict fold change MFI respect to unstimulated DCs **(B)** IL-6, IL-12p70, IFN-β, TNF, IL-10, IL-1β, and IL-18 production were detected by ELISA in the supernantant of DCs cultured with medium (empty bars) or FhCL3 (10 μg/ml), (blacks bars). **(C)** IL-1β and IL-18 secretion were evaluated in the supernatant of DCs with medium, FhCL3 (10 μg/ml) or rvFhCL3 (10 μg/ml) by ELISA. Data are presented as mean ± SD from at least four wells are representative results from three experiments. ***p < 0.001 (ANOVA test).

To make progress in the knowledge into the interaction between FhCL3 and DCs, we evaluated the ability of this protein to modify the maturation status of these cells. In the presence of FhCL3, immature DCs expressed increased levels of MHC class II molecules in comparison to untreated DCs (MFI fold increase FhCL3 vs. untreated DCs *p* < 0,001, Figure 1A), while the expression of co-stimulatory molecules CD40, CD80, and CD86 was not modified (Supplementary Figure 2A). FhCL3 stimulation of DCs did not induce IL-6, IL-12p70, IFN-β, TNF, or IL-10 production. Surprisingly, after this treatment, an increase in IL-1β and IL-18 secretion was detected as compared to untreated DCs (Figure 1B). Furthermore, the specific cysteine protease activity of FhCL3 was critical for IL-1β and IL-18 production, since the recombinant variant of FhCL3 (rvFhCL3), has a specific change in the amino acid sequence at the active site that abolishes FhCL3 unique ability to cut P_2_-P_3_ Pro-Gly residues but not completely inactivate the enzyme (25), it was unable to induce IL-1β and IL-18 secretion (Figure 1C).

IL-1β secretion depends on the synthesis of pro-IL-1β after canonical activation of NF-kB therefore, we wondered if FhCL3 could induce NF-kB activation. As it was previously reported, LPS induced p65 (30) and IkB-α (31) degradation between 10 and 30 min after DCs stimulation. However, the bands corresponding to p65 and IkB-α, were not modified between 10 and 30 min after FhCL3 treatment in comparison with unstimulated DCs (Supplementary Figure 2B), which indicates the lack of activation of NF-κB.

Altogether these data indicate that FhCL3 promotes the IL-1β and IL-18 release in DCs in the absence of priming or NF-κB activation. However, this effect depends on the enzymatic activity of FhCL3.

### FhCL3 Is Internalized by DCs and Targeted to the Cytosol

**Figure 2:**
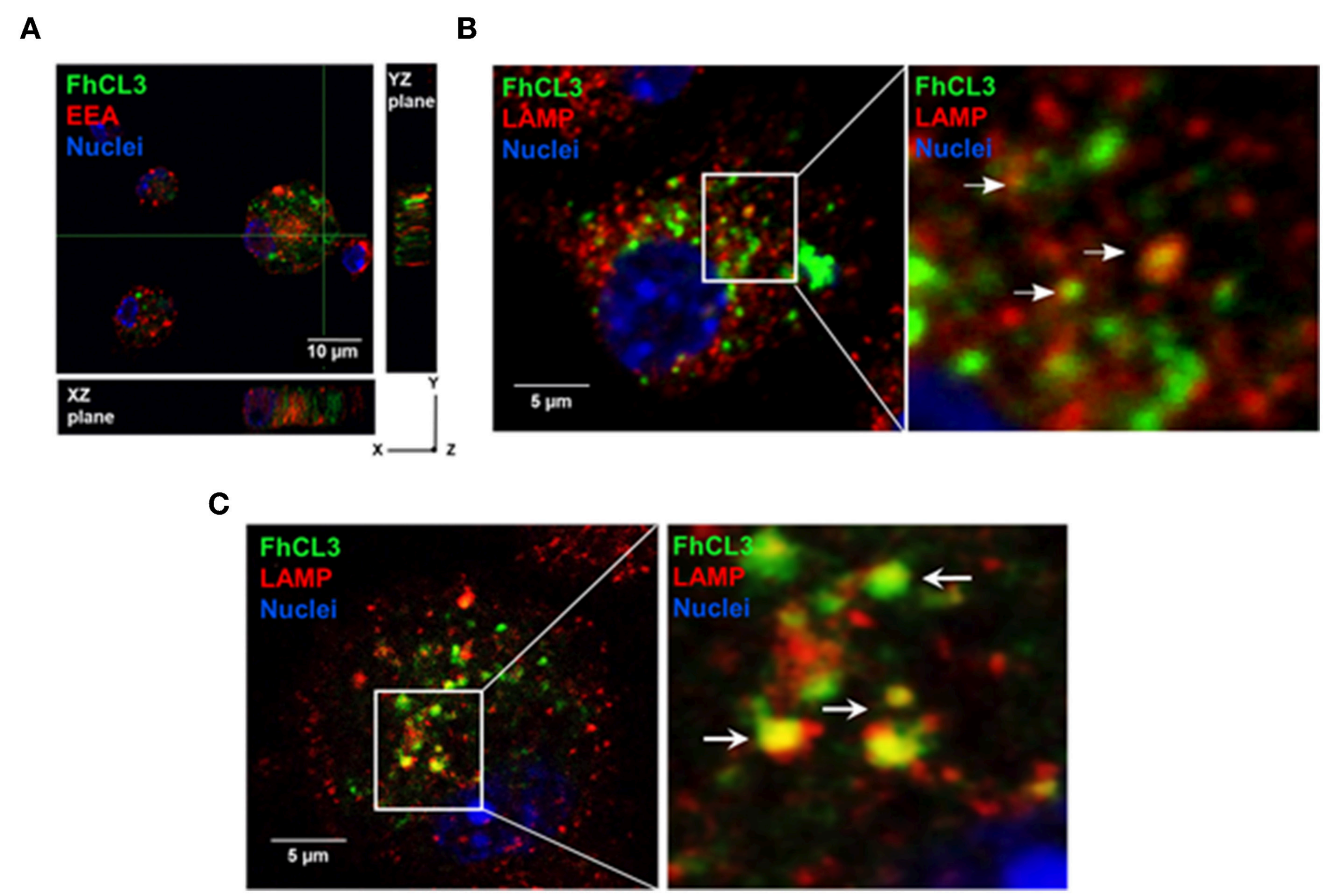
Intracellular location of FhCL3, analyzed by Confocal Laser Scanning Microscope. DCs were incubated for 30 min, 4 h or 18h with Alexa Fluor®488-labeled FhCL3 (green) to evaluate co-localization with early endosomes and lysosomes, respectively. **(A)** Cells were stained with EEA-1, early endosomal antigen 1, in red. Orthogonal projections of z-stacks are depicted. The cell nucleus was used as an internal reference point. The XZ and YZ planes show the cell height and width, and height and length, respectively. **(B,C)** LAMP, in lysosomes are showed red at 4 h and after 18 h, respectively. Cell Nuclei were stained with Hoechst. Higher magnifications of selected areas are shown on the right. These images are representative at least two experiments. The scale bar is 10 micron in **(A)** and 5 micron in **(B,C)**.

In order to evaluate whether FhCL3 is uptaked by DCs or not, these cells were treated with an Alexa fluor 488-labeled FhCL3 (Green). Confocal microscopy analysis revealed that after 2 h, labeled FhCL3 was internalized mostly to the cytosol, since the labeled-protein was located in early endosome antigen (EEA)-negative compartment (Figure 2A). In addition, a weak co-localization of FhCL3 with LAMP positive compartments was observed at 4 or 18 h of culture (Figures 2B,C).

We also observed that the level of Alexa fluor 488-labeled FhCL3 was substantially unaltered up to 18 h (Figure 2C). Series of confocal fluorescence pictures taken along the z-axis (z-stack) of labeled FhCL3-treated DCs, confirm the presence of green fluorescence (FhCL3) inside the cells (Figure 2A). These results show that FhCL3 is endocyted by DCs and remains unchanged for at least 18 h mainly in the cytosol of the cells.

### FhCL3 Induces the Expression, Maturation, and Release of IL-1β in the Absence of other Stimuli

**Figure 3:**
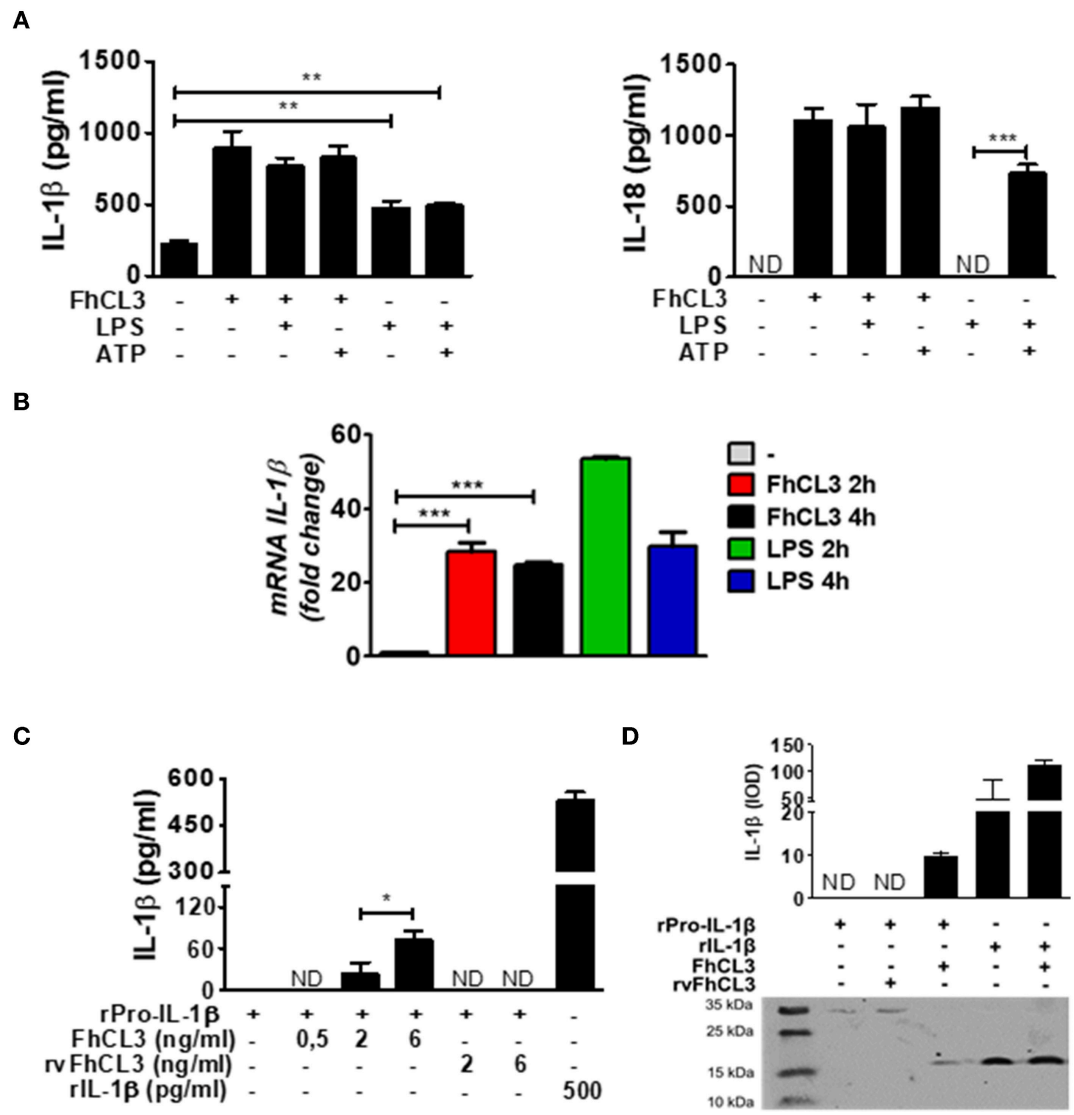
FhCL3 promotes the expression, proteolysis and secretion of IL-1β without LPS or ATP stimulation. **(A)** DCs were stimulated with medium, FhCL3 (10 μg/mL) and/or LPS (100 ng/mL) for 18 h; in same cases ATP (5 mM) was added the last 30 min of culture. IL-1β and IL-18 production were quantified by ELISA in culture supernatants. **(B)** DCs were treated with medium, FhCL3 (10 μg/mL) and/or LPS (100 ng/mL) for 2 or 4 h. mRNA levels of IL-1β were determined by q-PCR relative to the housekeeping gene β-actin and normalized to the level of unstimulated DCs. **(C)** Recombinant pro-IL-1β (31 kDa) was incubated at 37°C in sodium phosphate buffer, with or without FhCL3 (0.5, 2 or 6 ng/mL) or rvFhCL3 (2 or 6 ng/mL) for 2 h. Mature IL-1β levels were determined in supernatants by capture ELISA. Recombinant IL-1β (17 kDa) was used as positive control and represents the commonly detected mature form, whereas pro-IL-1β indicates the inactive pro-form. **(D)** Western blot illustrating the cleavage of pro-IL-1β by FhCL3 or rvFhCL3 (1 μg/mL). Recombinant IL-1β (17 kDa) or pro-IL-1β (37 kDa) were loaded as controls. The graph bars show the densitometry analysis of IL-1β (17 kDa) from two experiments with similar results. Bars panels show the mean ± SD and are representative of three or two independent experiments. ND, not detected; *p < 0.05; **p < 0.01; ***p < 0.001 (ANOVA test).

In order to know if FhCL3 is able to synergize with other stimuli causing the activation of the inflammasome, we cultivated DCs with FhCL3 in the presence or absence of LPS or ATP. The addition of LPS or ATP to FhCL3-stimulated DCs, did not modify IL-1β or IL-18 secretion compared to those produced by FhCL3-treated DCs (Figure 3A). As previously reported, upon LPS plus ATP DCs stimulation, an increase in IL-1β and IL-18 was observed (32), meanwhile the LPS stimulation only enhanced IL-1β production (Figure 3A). During inflammasome activation, the existence of a first signal has been reported as an essential step for the synthesis of the pro-form of IL-1β (among other components of the inflammasome), otherwise there would be no availability of substrate for caspase-1 to produce the active form of IL-1β (20, 33). Unlike IL-18, which is constitutively expressed in murine macrophages, dendritic cells, endothelial cells, intestinal epithelial cells, and keratinocytes under steady state (34), IL-1β requires an induction for its biosynthesis. This event is normally promoted via TLR ligands and other cytokines, leading to the synthesis of pro-IL-1β (35). We evaluate IL-1β mRNA expression by qPCR after 2 and 4 h of culture. Figure 3B shows that similarly to LPS, FhCL3 induces IL-1β gene transcription in DCs, compared to untreated cells (Figure 3B).

*In vitro* studies have shown that some proteases, such as neutrophil elastase, are able to process the pro-IL-1β into mature IL-1β (36). In addition, in the view of the fact that the enzymatic activity of FhCL3 was necessary to induce the biologically active form of IL-1β, we hypothesized that the FhCL3 cysteine protease activity, might be essential for the cleavage of pro-IL-1β. To evaluate this, we performed two approaches in a free cell system. First, a murine recombinant of IL-1β or pro-IL-1β was separately incubated with FhCL3 or rvFhCL3, in a slightly acid medium. At the end of the incubation time, IL-1β was detected by capture ELISA. The detection of mature IL-1β was possible in those wells where pro-IL-1β was treated with the enzymatically activate FhCL3. In contrast, in those cases where pro-IL-1β was treated with rvFhCL3, IL-1β was not detected (Figure 3C). In the second strategy, upon addition of FhCL3 to pro-IL-1β, a band corresponding to 17 kDa IL-1β form was detected by western blot, In contrast, when the rvFhCL3 was added to pro-IL-1β, the band corresponding to mature IL-1β, was not observed. Interestingly, the pro-IL-1β cleavage by FhCL3 seems to be site specific since the addition of FhCL3 to the mature IL-1 β form, did not modify the band corresponding to 17 kDa (Figure 3D). These data show an undescribed ability of FhCL3 to cleave pro-IL-1β through its cysteine protease activity.

### The Inflammasome Activation Induced by FhCL3 Is Dependent on NLRP3

Through inflammasome activation, the proteins of cytosolic multimeric complexes are assembled and mediate the maturation of IL-1β and IL-18. During inflammasome activation triggering, cytosolic NOD-like receptors (NLRs) are able to detect PAMPs or DAMPs, generating the active form of IL-1β and IL-18. Multiple molecules are able to activate NLRP3 inflammasome, among them there are PAMPS, bacterial toxins, ATP and a variety of crystal such as monosodium urate crystal (20).

**Figure 4:**
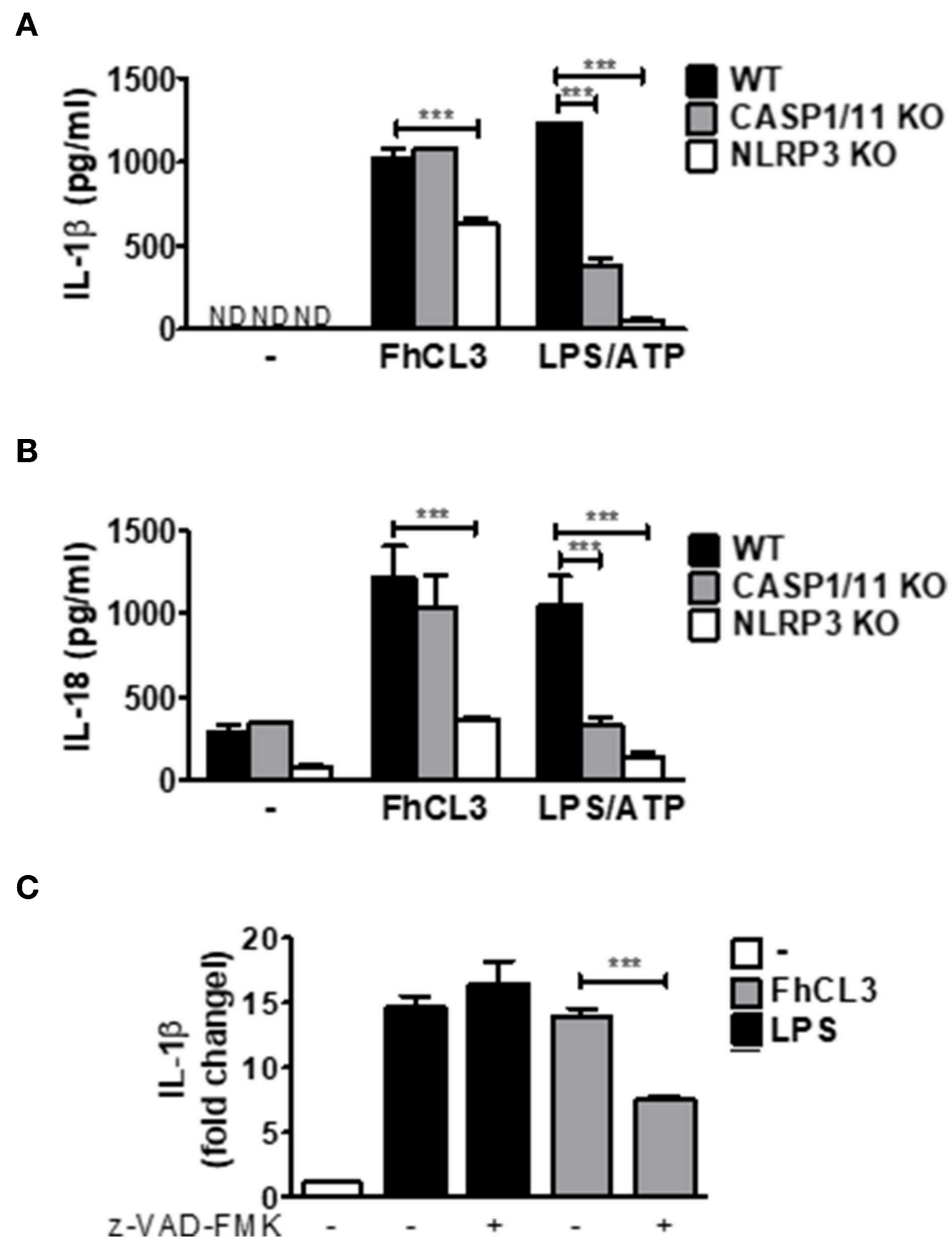
FhCL3 enhances IL-1β and IL-18 secretion in DCs by a NLRP3 dependent and caspase independent mechanism. **(A)** and **(B)** DCs from WT, CASP1/11 KO or NLRP3 KO mice were stimulated with medium or FhCL3 (10 μg/mL) for 18 h. In some cultures the cells were treated with LPS (100 ng/mL) for 18 h and ATP (5 mM) for the last 30 min of culture. IL-1β and IL-18 production were evaluated by ELISA in culture supernatants. Bars panels represent the mean ± SD from three independent assays ND, not detected; ***p < 0.001(ANOVA with Dunnett's post-test). **(C)** DCs pretreated or not with pan caspase inhibitor (Z-VAD-FMK, 5μM, 1h) were incubated with FhCL3 or LPS in the presence or absence of ATP during the last 30 min of a 18 h stimulation period. IL-1β secretion was quantified by ELISA in supernatants. The bars represent a ratio of IL-1β detected in culture supernatant of DCs with different stimuli vs. unstimulated DCs. Experiment was repeated three times with similar results being obtained and is expressed in bars graph, as the mean ± SD.

In order to evaluate if FhCL3 activates the NLRP3 inflammasome, *in vitro* culture using NLRP3-deficient DCs (NLRP3 KO) were performed. As shown in Figures 4A,B, in the absence of NLRP3 a partial, but significant inhibition of IL-1β and IL-18 production was observed in comparison to that produced by DCs from WT mice (*p* < 0.001 WT vs. NLRP3 KO mice). Since the bioactive molecule of IL-1β is available only after the cleavage of pro-IL-1β by caspase-1, a key effector molecule of the inflammasome, we tested its role in FhCL3-induced inflammasome activation. Unexpectedly, IL-1β and IL-18 secretion were not modified in FhCL3-treated DCs from CASP1/11KO (caspase 1/11 deficient mice), compared to that produced by WT DCs. In contrast, LPS/ATP-treated DCs from CASP1/11 KO or NLRP3 KO mice showed a significant decrease in IL-1β and IL-18 secretion (Figures 4A,B). Since some studies show that other caspases than 1 and 11, such as the caspases 4 and 5, are able to activate the NLRP3 inflammasome, we evaluated the production of IL-1β in FhCL3-treated DCs in the presence of the pan-caspase inhibitor Z-VAD-FMK. We observed that in spite of a complete inhibition of caspase activity, FhCL3 was still able to induce IL-1β production in DCs, corroborating the lack of involvement of caspases in this phenomenon (Figure 4C). Moreover, TNF and IL-12p70 production by LPS/ATP-stimulated DCs was unaffected by the absence of CASP 1/11 or NLRP3 (Supplementary Figures 3A,B). These results indicate that the inflammasome activation induced by FhCL3 is partially dependent on NLRP3, however, it is uncoupled from caspase activation.

### FhCL3 Induces IL-1β Through ROS Production, Potassium (K^+^) Efflux and Lysosomal Acidification

**Figure 5:**
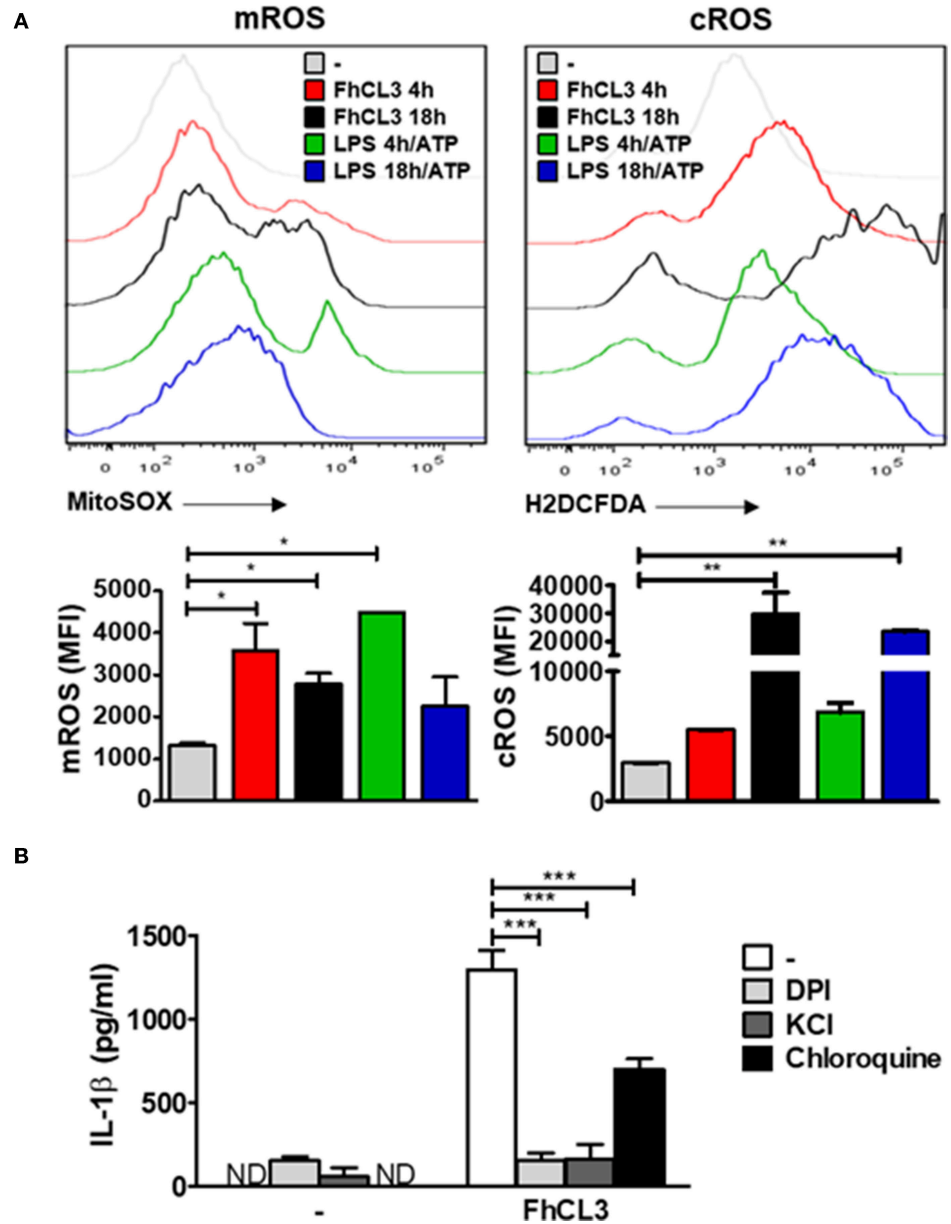
ROS production, potassium (K+) efflux and lysosomal acidification are involved in FhCL3-induced IL-1β production in DCs. **(A)** DCs were stimulated with medium, FhCL3 (10 μg/mL) or LPS (100 ng/mL) plus ATP (5 mM) for 4 or 18 h. ATP was added the last 30 min of culture. Cells were stained with anti-CD11c and anti-MHCII antibodies. Then, DCs were incubated with 5 μM MitoSOX probe (PE) or 20 μM H2DCFDA probe for 20 min at 37°C, and analyzed by Flow cytometry. Histograms represent mean fluorescence intensity (MFI) of mROS and cROS on CD11c+ MHCII+ gated populations with different treatments. Bars depict the mean ± SD and are representative of at least three independent experiments. **(B)** DCs pretreated or not with DPI (20 μM, for 3 h), KCl (50 mM, for 90 min) or choloroquine (5 mM for 1 h), were stimulated with FhCL3 (10 μg/mL) for 18 h. Then IL-1β secretion was quantified by ELISA in supernatants. Experiments were repeated three times with similar results being obtained and are expressed as in bars graph mean ± SD ND, not detected; *p < 0.05; **p < 0.01; ***p < 0.001 (ANOVA test).

Considering the fact that reactive oxygen species (ROS) production is a crucial event that triggers the inflammasome activation (37), we decided to evaluate its involvement in the FhCL3-induced DCs activation. To elucidate the role of these intermediary signals, DCs were treated with FhCL3 or LPS for 4 or 18 h, in the presence or absence of ATP during the last 30 min of culture, and the expression of mitochondrial ROS (mROS) and cytoplasmic ROS (cROS) was determined by flow cytometry. As shown in Figure 5A, FhCL3-conditioned DCs displayed an increase in the mean fluorescence intensity (MFI) of these cells expressing both mitochondrial (left panel) and cytoplasmic (right panel) ROS, when compared to the unstimulated control. The increase in mROS was observed at earlier times (4 h) in comparison to the cROS production (18 h). Similar results were observed in LPS-treated DCs for 4 h or 18 h followed by the addition of ATP (Figure 5A).

In order to study whether IL-1β production induced by FhCL3-treated DCs depends on ROS or not, we used a NADPH oxidase inhibitor, which is a critical enzyme involved in ROS production. First, DCs were pretreated with the NADPH oxidase inhibitor diphenyleneiodonium (DPI) for 30 min. Subsequently, the cells were stimulated with medium, FhCL3 or LPS/ATP. The inhibition of ROS production drastically reduced the amounts of IL-1β secreted by FhCL3-treated DCs (Figure 5B). Similar results were observed when DCs were treated with potassium chloride (KCl) to block potassium (K+) efflux, whereas a partial inhibition in IL-1β production was observed after the chloroquine treatment of DCs to inhibit lysosomal acidification (Figure 5B). These findings strongly suggest that ROS production, potassium (K+) efflux as well as lysosomal acidification are all events involved in FhCL3-induced IL-1β production in DCs.

### DCs Conditioned With FhCL3 Show Increased T-Cell Allostimulatory Capacity Promoting IFN-γ and IL-13 Responses

Then, we wanted to evaluate which T helper profile was promoted by FhCL3-treated DCs in a mixed lymphocyte reaction. To this end, FhCL3-treated DCs from C57BL/6 mice, were cultured with allogeneic splenocytes from BALB/c. Cultures with untreated DCs were used as control.

**Figure 6:**
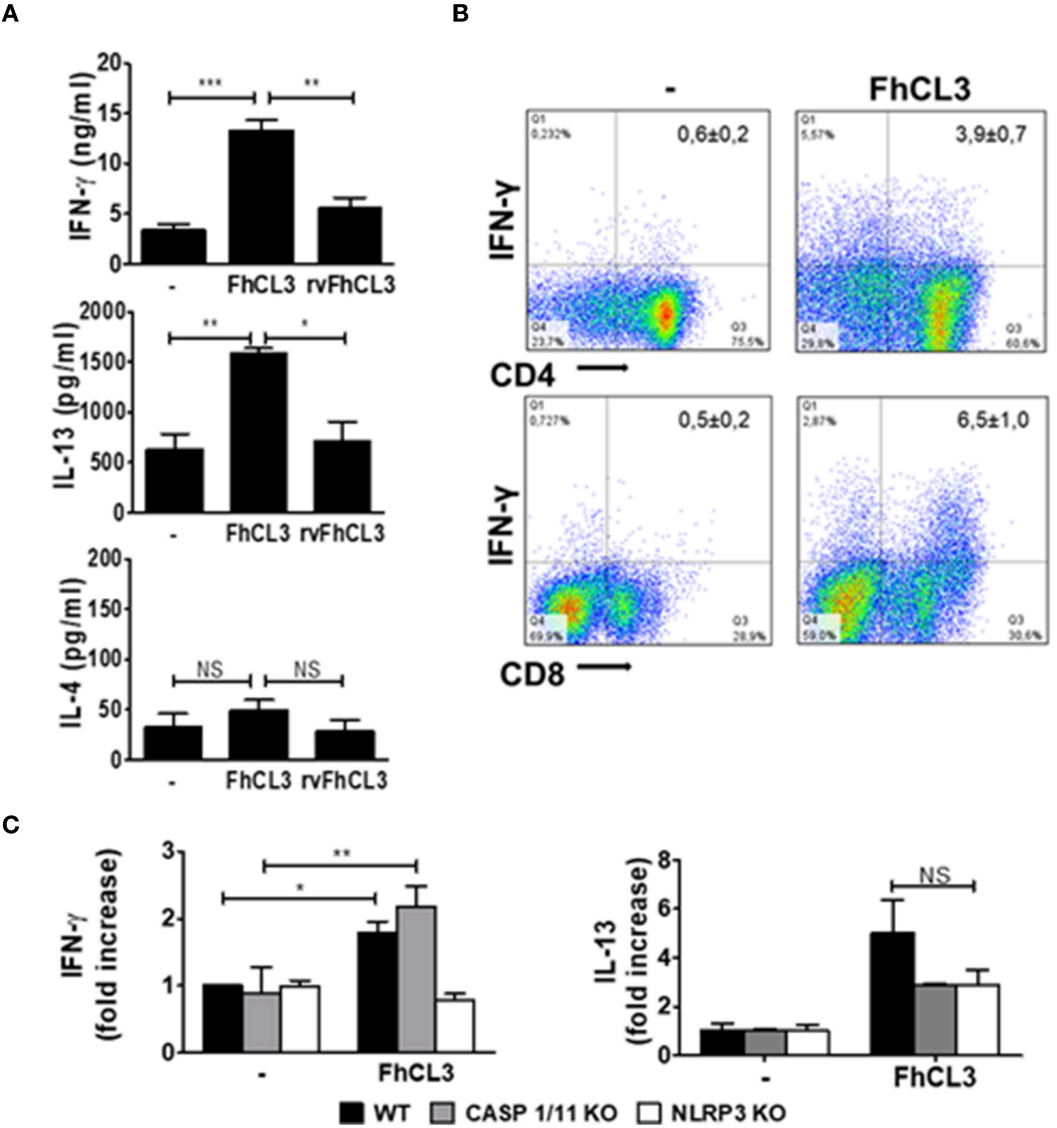
FhCL3-treated DCs induce an IFN-γ and IL-13 response in allogeneic cultures. DCs from WT, CASP1/11 KO or NLRP3 KO (all C57BL/6 background) were treated with medium, FhCL3 (10 μg/mL) or rvFhCL3 (10 μg/mL) for 18 h and co-cultured with allogeneic splenocytes from BALB/c mice. **(A)** At day 5, IFN-γ, IL-4 and IL-13 concentrations were measured in supernatants by ELISA. **(B)** The splenocytes were stimulated with PMA/Ionomycin for 4 h and analyzed by flow cytometry. IFN-γ expression was evaluated on CD4+ and CD8+ T cells on CD3+ gated population by flow cytometry. Data are showed in representative dot plot diagram of three or four independent experiments. In the upper quadrants the values indicate the percentages as mean ± SD. **(C)** IFN-γ and IL-13 production were determined in culture supernatants by ELISA. The concentrations were normalized according to a ratio of IFN-γ or IL-13 detected in culture supernatant of allogeneic splenocytes cultured with FhCL3-treated DCs vs. unstimulated DCs. Results are presented in bars graph as means ± SD and are representative of three experiments. NS, not significant, *p < 0.05; **p < 0.01; ***p < 0.001 (ANOVA test).

After 18 h of culture, increased amounts of IFN-γ and IL-13 were detected in the supernatant of allogeneic cultures when DCs were stimulated with FhCL3. However, the levels of these two cytokines were not modified after rvFhCL3-treated DCs stimulation (Figure 6A). Besides, no changes in IL-4 production were detected in allogeneic cultures with FhCL3-treated DCs (Figure 6A). The analysis of allogeneic splenocytes after cultured with FhCL3-treated DCs by flow cytometry showed an increase in T CD4 (from 0.6 to 3.9%) and T CD8 (from 0.5 to 6.5%)

IFN-γ expressing cells, compared to the response of splenocytes incubated with untreated DCs (Figure 6B). Similar results were observed with DCs from BALB/c mice pulsed with FhCL3 in cultures with splenocytes from C57BL/6 animals (data not shown).

Due the involvement of NLRP3 in FhCL3-induced DCs inflammasome activation, we evaluated the T cell allostimulatory capacity of DCs from NLRP3 KO mice. In agreement with the results showed above, the ability of FhCL3-treated DCs to prime T cells response diminished in the absence of NLRP3, since IFN-γ level was significantly lower than that induced by DCs from WT mice (Figure 6B). In contrast, DCs from CASP1/11 KO mice were able to induce similar levels of IFN-γ production by splenocytes in cultures with DCs from WT mice (Figure 6C). The capacity of FhCL3-treated DCs from NLRP3 or CASP1/11 KO to induce IL-13 secretion by allogeneic splenocytes was similar to that produced in allogeneic cultures with WT DCs (Figure 6C).

These data indicate that DCs stimulated with FhCL3 prime splenocytes toward a singular profile characterized by high levels of IFN-γ and IL-13, with this capacity depending on the cysteine protease activity of FhCL3. In addition, the ability to induce IFN-γ of FhCL3-conditionated DCs is independent of caspases 1/11 and dependent on NLRP3, suggesting a non-canonical activation of the inflammasome.

### FhCL3-treated DCs Induce Increased IFN-γ Secreting T Cells *in vivo*

**Figure 7:**
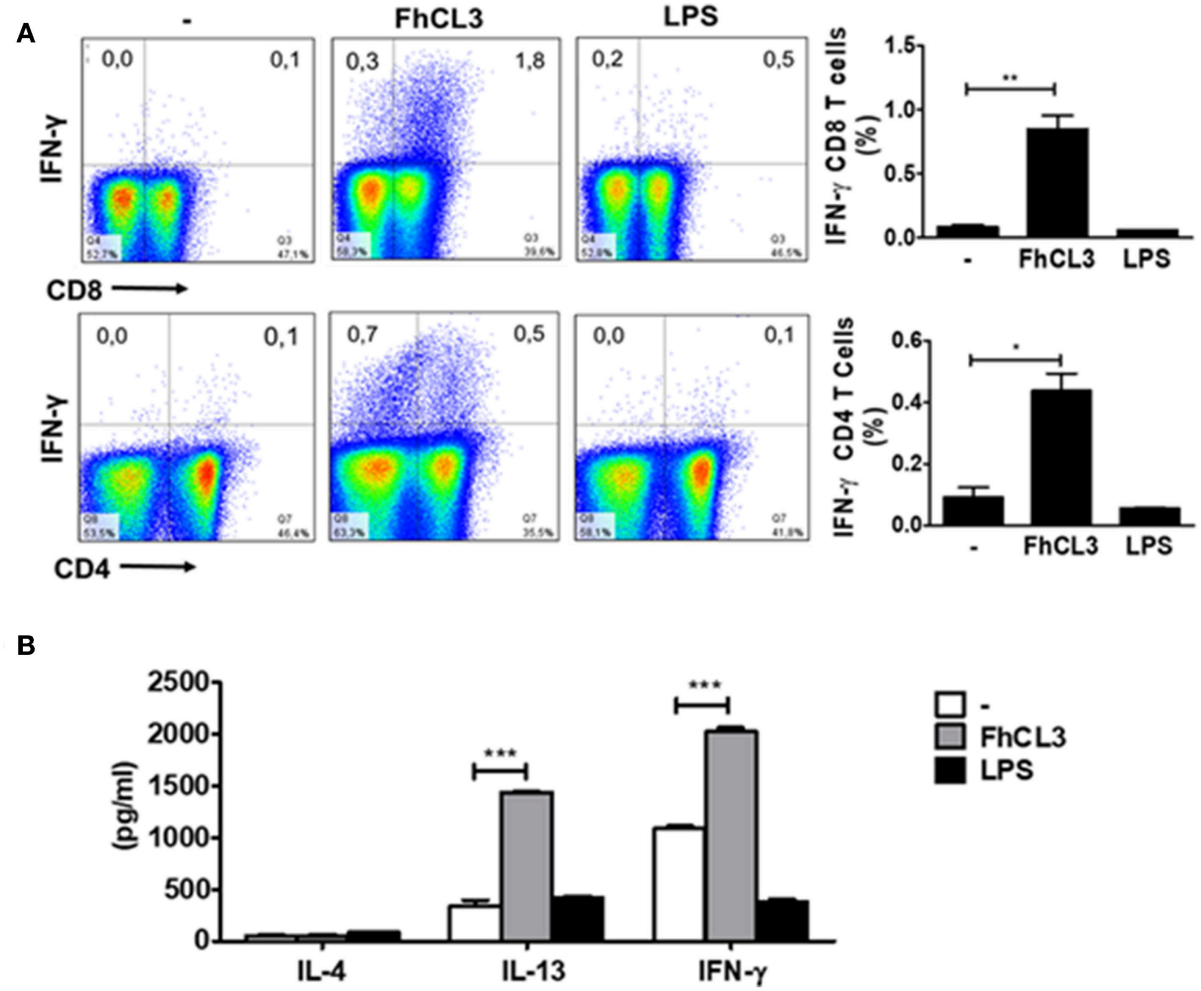
FhCL3-treated DCs promote a specific IFN-γ response *in vivo*. C57BL/6 mice were injected intraperitoneally with DCs treated or not with FhCL3 or LPS. At day 7, cells from iLN were obtained and re-stimulated with FhCL3 for 48 h. **(A)** The cells were stimulated with PMA/Ionomycin for 4 h and analyzed by flow cytometry. The IFN-γ production was evaluated in CD4+ and CD8+ T cells on CD3+ gated population from inguinal lymph node. Data of flow cytometry are showed in representative dot plot diagram of three independent experiments. To the right, bar graphs depict the frequency of gated T cells. **(B)** The IL-4, IL-13 and IFN-γ production were evaluated in culture supernatant by ELISA. Results are presented in bars graph as means ± SD and are representative of three experiments. *p < 0.05; **p < 0.01; ***p < 0.001 (ANOVA test).

Next, we explored the ability of FhCL3-treated DCs to induce a specific T cell response in lymph node of mice injected with these cells. After 7 days of intraperitoneally administration, iLN were removed, and suspensions of lymphocytes were restimulated with FhCL3 for 48 h. Significantly increased levels of IFN-γ and IL-13 were detected in the supernatant of iLN cultures of mice injected with FhCL3-treated DCs (Figure 7A). Additionally, mice injected with FhCL3-treated DCs, mounted a specific CD8-IFN-γ expressing T cell response and to a lesser extent.

CD4-IFN-γ in T cells present in iLN (Figure 7B), in comparison with cells from iLN of mice injected with untreated DCs. In contrast, mice injected with LPS-stimulated DCs, failed to develop a specific IFN-γ against FhCL3 (Figure 7B). These data indicate that FhCL3-treated DCs can prime T cells from iLN *in vivo* toward a specific response characterized by IFN-γ and IL-13 cytokine profile.

## Discussion

IL-1β is synthesized as an inactive form of 31 kDa precursor, termed pro-IL-1β, after recognition of PAMPs. Processing of bioactive IL-1β (and that of IL-18) depends on caspase-1 activation by protein complexes called inflammasomes (38). The activity of cysteine protease of caspase-1 raised the question of whether other enzymes coming from cells or microorganisms have the ability to process pro-IL-1β or not. Indeed, some studies have showed that neutrophil- and macrophage-derived serine proteases such as a neutrophil serine protease, proteinase-3, elastase, and cathepsin-G enzymes are able to process pro-IL-1β into 17 kDa active fragment (39, 40). In addition, matrix metalloproteinases (MMP), such as stromelysin 1, gelatinases A and B, have also shown the ability to cleave pro-IL-1β (41). Similarly, studies using either blastospores or hyphae from *Candida albicans* in the presence of proteinase inhibitors, revealed that the cleavage of pro-IL-1β involves the participation of aspartyl proteinases (42). Interestingly, the ability to induce IL-1β production in the infection with M*ycobacterium tuberculosis*, showed the existence of alternative pathways of IL-β and IL-18 activation by a mechanism that does not require TLR signaling or caspase-1(43). Despite these reports, much less information is available about the capability of helminth-derived proteases to modulate inflammasome activation.

In the present study, it was the first time that a cysteine protease from a helminth parasite was found to induce an inflammasome alternative activation, which causes the production of IL-1β and IL-18 in DCs. This statement is based on experiments showing that FhCL3 did not follow the conventional steps of activation described for the IL-1β synthesis during innate immune responses toward different pathogens. It is well-known that two signals are required to induce inflammasome activation: the signal one, induced by PAMPs or DAMPs derived from tissue damage, which in turn activates innate immunity receptors such as TLR and lead to the synthesis of pro-IL-1β depending on NF-kB activation (20). The induction of IL-1β by FhCL3 treatment, did not require priming signals since the mere presence of the enzyme was a sufficient stimulus to increase the production of this cytokine, and did not change when LPS or ATP was added to the DCs cultures. Despite the independence of NF-kB in FhCL3-induced DCs activation, the expression of the gene coding for IL-1β increased more than 20 times, in relation to the basal condition in unstimulated DCs. Interestingly, these findings are consistent with a recent study which has demonstrated that endogenous cathepsins are implicated in the nigericin-induced inflammasome activation, since they can induce both: pro-IL-1β synthesis and NLRP3 activation (44). The lack of involvement of NF-kB in FhCL3-induced DCs activation, suggests that other/s transcription factor might be involved in the synthesis of pro-IL-1β. In this respect, either IFN regulatory factors IRF-4 or IRF-8 has been involved in the IL-1β transcription and more recently the kinase CK2 has been described as a master regulator of two independent pathways that promote IL-1β transcription, one of the pathway was dependent on NF-kB p65 and the other dependent on IRF4 activation (43). More experiments should be performed, in order to evaluate possible candidates that mediate FhCL3-induced pro-IL-1β synthesis.

The second signal of inflammasome activation, promotes the oligomerization of the receptors belonging to the NOD-like receptors (NLRs) family, being the most studied the pyrin domain-containing protein 3 or NLRP3 (45, 46). The subsequent assembly of NLRP3, ASC and procaspase-1 into a complex, constitute the scaffold that mediates the generation of inflammatory caspases. The latter is involved in the processing of pro-IL-1β and the pro-IL-18 toward its active forms. The potassium efflux (47), the lysosomal disruption that leads to release of cathepsin B into the cytosol (48) and the intracellular generation of reactive oxygen species (ROS) (49) are among the signals capable of inducing the assembly of the NLRP3 inflammasome. In this work, the inhibition of ROS production, potassium efflux and the lysosomal acidification caused a decrease in the IL-1β secreted by FhCL3-treated DCs, which suggests the involvement of these mediators in inflammasome activation. However, the ability of activated DCs to produce IL-1β was not dependent on caspases. This ability was demonstrated by the use of DCs from deficient mice in caspases 1 and 11, as well as the pan caspase inhibitor Z-VAD-FMK, which was able to block other caspases such as 4 and 5 involved in inflammasome activation, suggesting the involvement of other enzyme different to caspase in the cleavage of pro-IL-1β. This hypothesis, was clearly demonstrated when an inactive recombinant form of FhCL3 was unable to induce IL-1β production in DCs. These data were confirmed in a free cell system in which an active recombinant form of FhCL3 was capable to cleave a pro-IL-1β recombinant to its active form of IL-1β that was detected by ELISA and western blot.

After being endocyted, helminth molecules, such as SEA of *S. mansoni* and the filarial antigen ES-62, are located in LAMP1+ vesicles on DCs and macrophages, respectively (29, 50). However, only a weak colocalization with lysosomes was evidenced after endocytosis of FhCL3, which persisted within the cells at least 18 h post-incubation. It has been described that FhCL1 is internalized by peritoneal macrophages, and unlike FhCL3, presents a greater localization within early endosomes. Due to the poor colocalization of FhCL3 with endosomes or lysosomes, the mechanism of endocytosis could be different from that used by FhCL1. Considering that FhCL3 is a molecule of low molecular weight, the entry into the cell could be mediated by a mechanism of pinocytosis. However, the use of a pinocytosis inhibitor (cytochalasin D), in cultures of DCs treated with FhCL3, did not affect either the entrance of the cells, or the production of IL-1β (data not shown), suggesting that FhCL3 could be endocyted trough a receptor pathway.

Even though FhCL3 induces an increase in the production of cytokines considered as pro-inflammatory such as IL-1β or IL-18, it does not generate a fully activation on DCs, since other typical inflammatory cytokines as well as co-stimulatory molecules were not increased. Meanwhile only a partial up-regulation of MHCII was observed. This increase could be explained by a mechanism that correlates IL-1β secretion and the release of the MHCII to the cell membrane, being these events absolutely necessary to the inflammasome assembly (51)

The involvement of NLRP3 in the FhCL3-induced DCs IL-1β production, could be explained in two different ways: on the one hand, a direct interaction between FhCL3 and NLRP3 could occur during the inflammasome activation. Further experiments should be performed to confirm this hypothesis. On the other hand, ROS production, potassium efflux and lysosomal disruption might act as intermediaries in the activation of NLRP3 inflammasome. These data, together with the cytosolic location, suggest that FhCL3 would have a caspase like activity in DCs during the inflammasome activation. In contrast, data published by Alvarado et al. show that FhHDM-1, a cathelicidin-like peptide secreted by *F. hepatica*, inhibits the inflammasome activation by a mechanism depending on lysosomal acidification (23). Within the 24 h after the infection with *F. hepatica*, the recruitment of alternative macrophages (M2) in the peritoneum of infected animals is induced by a predominant Th2 response (52, 53). However, an excessive response could be deleterious for the parasite. The IL-1β secretion, which controls the innate cytokines IL-25 and IL-33 production, both critical for the Th2 promotion, has been proposed as a mechanism to assure the chronicity of the parasite in intestinal infections by nematodes. A similar phenomenon might be possible during fasciolosis, since FhCL3 expressed by the juvenile worm could induce IL-1β, while FhHDM-1 secreted in all the stages (54) would control the function of the inflammasome. FhHDM-1, together with Fh12 (55), thioredoxin peroxidase (52) and kunitz type molecule (10), among others inhibitory molecules, could lead to a permanent suppression of inflammation during *F. hepatica* infection. *In vitro* experiments trying to dissect how FhCL3 and FhHDM-1 regulate the function of the inflammasome should be performed. *In vivo* silencing of FhCL3 using RNA interference in the infective stage, can causes a reduction in the penetration capacity of juvenile larvae through the intestinal wall interfering the life cycle of the parasite (56).

The treatment of the DCs with FhCL3, conditions these cells to stimulate the production of IFN-γ and IL-13 in allogeneic splenocytes. In this work, it was established that the main source of IFN-γ were CD8 T lymphocytes and to a greater extent, CD4 T cells. The dependence of this inflammasome was determined by the reduced ability of NLRP3 deficient DCs to promote the production of IFN-γ in cultures with allogeneic splenocytes. These results are in line with evidences showing that the deficiency of NLRP3 in mice infected with *Trichuris muris*, correlates with a reduced IFN-γ response, increased type 2 responses, and consequently the worm expulsion is accelerated (57). These data highlight the important role of NLRP3 in limiting protective immunity to this helminth.

The lack of dependence between NLRP3 inflammasome activation in DCs and the polarization of the response of IL-13 in allogenic lymphocytes, could be due to the opposing functions of IL-1β and IL-18 on the synthesis of IL-13. While some reports show the involvement of IL-18 in the up-regulation of IL-13 (58), others indicate an inhibitory role of IL-1β (13).

A question that arises from the comparison between the inflammasome activation of antigen presenting cells (APC) after infection with intracellular pathogens such as bacteria or protozoa and the inflammatory activation produced by extracellular pathogens such as helminths, is the fate of the APC after the inflammatory process (59). In contrast, very little information is available about the destination of the cells after inflammasome activation in helminth infections. In this respect, in our work we did not observe an increase in LDH, which is an enzyme released as a measure of pyroptosis (60), in FhCL3-treated DCs. In agreement with these data, Alvarado et al. have shown the ability of FhHDM-1 to diminish LDH release in LPS stimulated macrophages, which suggest that these cells would not die by pyroptosis.

In summary, this is the first report that describes a molecule derived from *F. hepatica*, FhCL3, with the ability to promote an alternative activation of the NLRP3 inflammasome, independently of caspase activity, which is characterized by the release of both, IL-1β and IL-18. These events have consequences on the adaptive immune responses, given that FhCL3-treated DCs promote a signature profile pattern of cytokines IFN-γ and IL-13 that could turn out to be protective in this parasitosis. Thus, this study may provide useful information for vaccines designing.

## Data Availability

All datasets generated for this study are included in the manuscript and/or the Supplementary Files.

## Author Contributions

LC, DC, and CM conceived and designed the experiments. DC, IC, LS, and AA performed the experiments. LC, DC, CM, IC, and MF analyzed the data. LC, DC, CM, IC, JT, and LSC contributed reagents, materials, analysis tools. LC, DC, CM, and LSC wrote the paper.

### Conflict of Interest Statement

The authors declare that the research was conducted in the absence of any commercial or financial relationships that could be construed as a potential conflict of interest.

## Supplementary Material

Supplementary Figure 1 FhCL3 biochemical features. **(A)** Determination of FhCL3 activity by hydrolysis of Tos-GPR-AMC. Initial rates were measured at 60-s intervals for 20 min and plotted as Relative Fluorescence Units (RFU) of AMC released from substrate per unit of time (min). **(B)** Mass spectrometric analysis by matrix-assisted laser desorption ionization time-of-flight mass spectrometry (MALDI-TOF MS) of FhCL3. The recombinant analysis identifies two peaks correspond to 29 kDa and 38,6 kDa consistent with molecular mass of the active enzyme and proenzyme, respectively.

Supplementary Figure 2 FhCL3 does not modify the expression of co-stimulatory molecules and NF-κB activation. **(A)** DCs were cultured with medium, FhCL3 (10 μg/mL) or LPS (100 ng/mL) for 18 h. Expression level of MHCI and co-stimulatory molecules CD40, CD80, and CD86 were evaluated by flow cytometry. Data are showed in representative histogram of three independent experiments. Values indicating mean fluorescent intensity (MFI) and percentage of expression. Below, bar graphs depict fold change MFI respect to unstimulated DCs. Bar graphs shown means ± SD (ANOVA test ^*^*p* < 0.05; ^**^*p* < 0.01; ^***^*p* < 0.001). **(B)** DCs were stimulated with medium, FhCL3 or LPS for 10 or 30 min. p65 and IkB-α expression were evaluated by western blotting. The blot is representative of two experiments with similar results.

Supplementary Figure 3 (A,B) DCs from WT, CASP1/11 KO or NLRP3 KO mice were stimulated with medium or FhCL3 (10 μg/mL) for 18 h. In some cultures the cells were treated with LPS (100 ng/mL) for 18 h and ATP (5 mM) for the last 30 min of culture. TNF and IL-12p70 production were evaluated by ELISA in culture supernatants. Bars panels represent the mean ± SD from three independent assays (ND, not detected; ANOVA with Dunnett's post-test).
