# Supplementary figures and images for: Cathepsin L3 From Fasciola hepatica Induces NLRP3 Inflammasome Alternative Activation in Murine Dendritic Cells
Source: Front Immunol. 2019 Mar 22;10:552. doi: 10.3389/fimmu.2019.00552 (PMC6438957; doi:10.3389/fimmu.2019.00552)

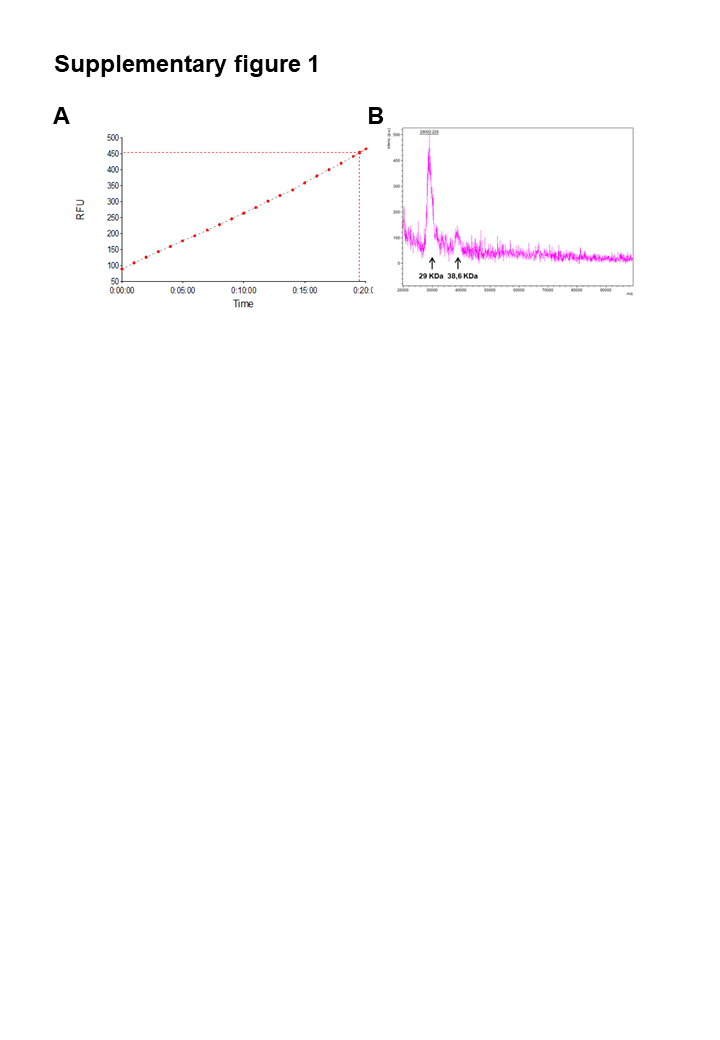

Supplement: Supplementary Figure 1 — FhCL3 biochemical features. (A) Determination of FhCL3 activity by hydrolysis of Tos-GPR-AMC. Initial rates were measured at 60-s intervals for 20 min and plotted as Relative Fluorescence Units (RFU) of AMC released from substrate per unit of time (min). (B) Mass spectrometric analysis by matrix-assisted laser desorption ionization time-of-flight mass spectrometry (MALDI-TOF MS) of FhCL3. The recombinant analysis identifies two peaks correspond to 29 kDa and 38,6 kDa consistent with molecular mass of the active enzyme and proenzyme, respectively. [file Image_1.TIF]

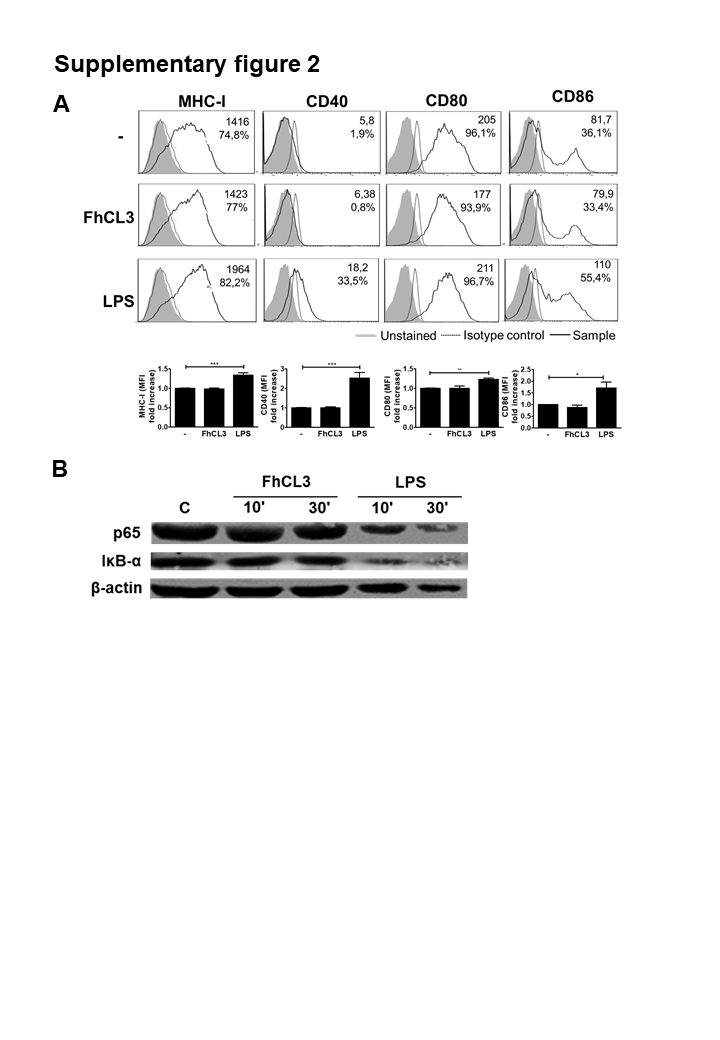

Supplement: Supplementary Figure 2 — FhCL3 does not modify the expression of co-stimulatory molecules and NF-κB activation. (A) DCs were cultured with medium, FhCL3 (10 μg/mL) or LPS (100 ng/mL) for 18 h. Expression level of MHCI and co-stimulatory molecules CD40, CD80, and CD86 were evaluated by flow cytometry. Data are showed in representative histogram of three independent experiments. Values indicating mean fluorescent intensity (MFI) and percentage of expression. Below, bar graphs depict fold change MFI respect to unstimulated DCs. Bar graphs shown means ± SD (ANOVA test *p < 0.05; **p < 0.01; ***p < 0.001). (B) DCs were stimulated with medium, FhCL3 or LPS for 10 or 30 min. p65 and IkB-α expression were evaluated by western blotting. The blot is representative of two experiments with similar results. [file Image_2.TIF]

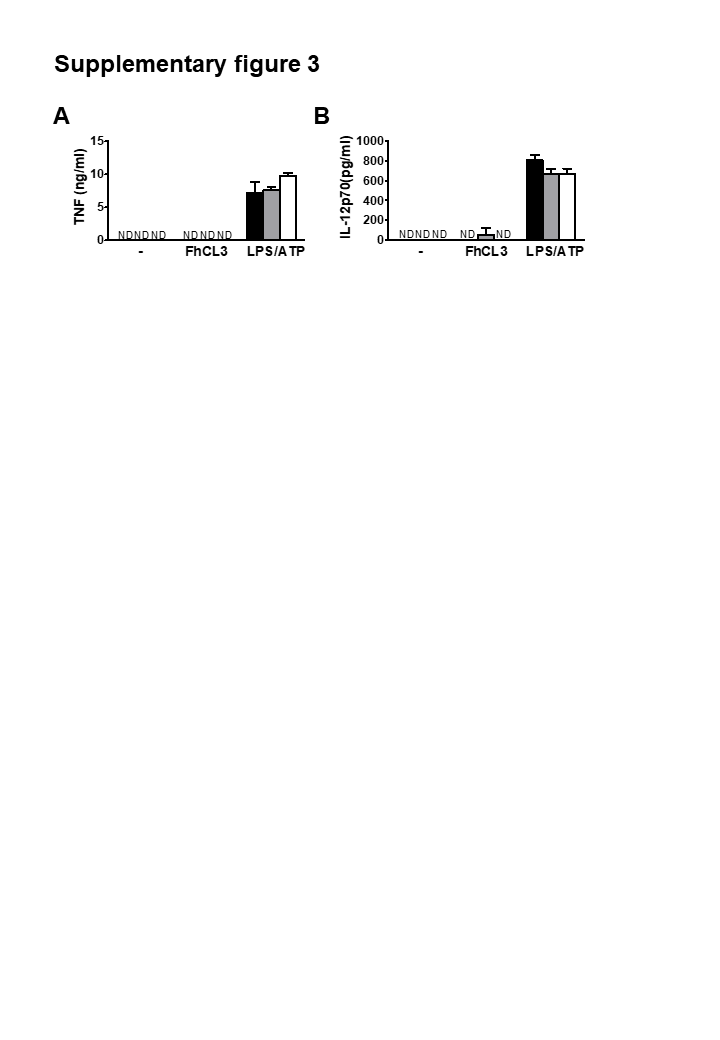

Supplement: Supplementary Figure 3 — (A,B) DCs from WT, CASP1/11 KO or NLRP3 KO mice were stimulated with medium or FhCL3 (10 μg/mL) for 18 h. In some cultures the cells were treated with LPS (100 ng/mL) for 18 h and ATP (5 mM) for the last 30 min of culture. TNF and IL-12p70 production were evaluated by ELISA in culture supernatants. Bars panels represent the mean ± SD from three independent assays (ND, not detected; ANOVA with Dunnett's post-test). [file Image_3.TIF]
